# Differential Shannon Entropies Characterizing Electron–Nuclear Dynamics and Correlation: Momentum-Space Versus Coordinate-Space Wave Packet Motion

**DOI:** 10.3390/e25070970

**Published:** 2023-06-23

**Authors:** Peter Schürger, Volker Engel

**Affiliations:** Institute of Physical and Theoretical Chemistry, University of Würzburg, Emil-Fischer-Str. 42, 97074 Würzburg, Germany; volker.engel@uni-wuerzburg.de

**Keywords:** differential Shannon entropy, correlation, electron–nuclear dynamics

## Abstract

We calculate differential Shannon entropies derived from time-dependent coordinate-space and momentum-space probability densities. This is performed for a prototype system of a coupled electron–nuclear motion. Two situations are considered, where one is a Born–Oppenheimer adiabatic dynamics, and the other is a diabatic motion involving strong non-adiabatic transitions. The information about coordinate- and momentum-space dynamics derived from the total and single-particle entropies is discussed and interpreted with the help of analytical models. From the entropies, we derive mutual information, which is a measure for the electron–nuclear correlation. In the adiabatic case, it is found that such correlations are manifested differently in coordinate- and momentum space. For the diabatic dynamics, we show that it is possible to decompose the entropies into state-specific contributions.

## 1. Introduction

Given the coordinate-space wave function ψ(x,t) of a quantum system, the differential Shannon entropy is obtained from the probability density $\rho \left(x,t\right)={\left|\psi \left(x,t\right)\right|}^{2}$ as [1,2]

(1) $$\begin{array}{c} S_{x}\left(t\right)=-\int dx\;\rho \left(x,t\right)\;\ln\left[\rho (x,t)\right], \end{array}$$

where *x* and *t* stand for the coordinates and time, respectively. This function is a measure for the information available on the system, and the larger its value, the less information is provided. Likewise, one may start with the momentum (π) space wave functions ψ(π,t) being the Fourier transform of ψ(x,t), yielding the density $\rho \left(\pi ,t\right)={\left|\psi \left(\pi ,t\right)\right|}^{2}$, and define the entropy

(2) $$\begin{array}{c} S_{\pi }\left(t\right)=-\int d\pi \;\rho \left(\pi ,t\right)\;\ln\left[\rho (\pi ,t)\right]. \end{array}$$

Following the information-theoretical line of thought, $S_{x}\left(t\right)$ and $S_{\pi }\left(t\right)$ provide us with knowledge about what happens in coordinate- and momentum-space, respectively. If one encounters a less localized coordinate-space probability density, the position of a particle is less precisely known so that the entropy $S_{x}\left(t\right)$ takes on a larger value. If we consider, for example, a Gaussian-like density, due to the Fourier relation, a broad coordinate-space distribution is associated with a more localized momentum-space density and, accordingly, $S_{\pi }\left(t\right)$ is smaller. This general behavior is connected to the coordinate-momentum uncertainty relation. In more detail, one finds that the sum $S_{x}\left(t\right)+S_{\pi }\left(t\right)$ is a measure for the coordinate-momentum uncertainty [2,3,4,5].

Concerning chemical dynamics, differential Shannon entropies have been discussed, see Refs. [6,7,8,9,10]. They are also important in the connection with reactivity [11], aromaticity [12] and stereochemistry [10]. Other applications include the thermalization of isolated quantum systems caused by disorder [13,14], and in the static case, differential Shannon entropies were applied to study wavefunction behavior in various potentials [15,16,17], avoided crossings [18,19,20] and correlation effects [21,22].

Regard now, more specifically, a molecule composed of electrons and nuclei. We then may calculate the differential Shannon entropies $S_{x}\left(t\right)$ and $S_{\pi }\left(t\right)$ from the total probability densities; additionally, using the electron (el) and nuclear (nuc) densities, the particle entropies $S_{x}^{el}\left(t\right),S_{\pi }^{el}\left(t\right)$ and $S_{x}^{nuc}\left(t\right),S_{\pi }^{nuc}\left(t\right)$ are accessible. It is the purpose of this paper to illustrate coordinate-space and momentum-space entropies for a coupled electron–nuclear dynamics. There, one may distinguish two opposite situations. The first one is that of a Born–Oppenheimer (BO) adiabatic motion [23,24], where the nuclear dynamics is restricted to a single electronic state, and couplings to other states are negligible. This is often realized if the motion takes place in the electronic ground state. The opposite limit is reached if strong non-adiabatic couplings are present [25]. Then, nuclear densities are transferred with large efficiency between different electronic states, as is usually the case when an avoided crossing between potential curves [26,27] or a conical intersection between potential surfaces [28,29,30] is passed. Differential entropies evolving from the weak and strong coupling cases are considered in this paper. Additionally, the electron–nuclear correlation, which can be characterized by the “mutual information” derived from the entropy functions [21,31], is discussed. In doing so, the interpretations evolving from coordinate-space and momentum-space are investigated, thereby extending our former work [32]. This is performed using analytical approaches and also giving numerical examples. The latter are restricted to a coupled one-dimensional motion of an electron and a nucleus [33,34]. We use parameterizations of the electronic–nuclear interaction potential, which cover the two coupling cases outlined above. The paper is organized as follows. In Section 2, we describe the model used in the numerical calculations, and we also provide the basic equations to arrive at the various entropies. The analytical and numerical results are collected in Section 3, and a summary is given in Section 4.

## 2. Theory and Model

### 2.1. Model for the Coupled Electronic–Nuclear Motion

A useful model for the one-dimensional electron–nuclear motion was established in the work of Shin and Metiu [33,34]. It has been used to describe basic properties of such dynamics [35,36,37], and was extended to include more than one electron [36] and also a planar motion to describe dynamics taking place at a conical intersection [38,39,40]. The interaction potential is taken as (in atomic units)

(3) $$\begin{array}{ccc} & & V\left(r,R\right)=\frac{1}{|R_{1}-R|}+\frac{1}{|R_{2}-R|}-\frac{erf[|R_{1}-r|/R_{f}]}{|R_{1}-r|}\\ & & -\frac{erf[|R-r|/R_{c}]}{\left|R-r\right|}-\frac{erf[|R_{2}-r|/R_{f}]}{|R_{2}-r|}+\Delta , \end{array}$$

where *r* and *R* denote the coordinate of the electron and nucleus, respectively. They interact via screened Coulomb potentials involving error functions. Additionally, there are two protons at fixed positions $R_{1}$ = − 5 Å and $R_{2}$ = + 5 Å so that further terms are present in the potential energy surface. The strength of the screening is determined by the two parameters $R_{f}$ and $R_{c}$, where in our examples, $R_{f}$ is fixed at a value of 1.5 Å, and $R_{c}$ takes the value of $R_{c}$ = 1 Å and $R_{c}$ = 5 Å for the cases of weak and strong non-adiabatic coupling, respectively. Finally, Δ is an energy shift which is chosen such that the potential V(r,R) has its minimum at zero energy in the range of our spatial grid.

The time-dependent Schrödinger equation reads

(4) $$\begin{array}{c} i\hbar \frac{\partial }{\partial t}\Psi \left(r,R,t\right)=\hat{H}\;\Psi \left(r,R,t\right), \end{array}$$

with the Hamiltonian

(5) $$\begin{array}{c} \hat{H}=\frac{{\hat{p}}^{\;2}}{2m_{e}}+\frac{{\hat{P}}^{\;2}}{2M}+V\left(r,R\right). \end{array}$$

Here, the momentum operators for the electron and the nucleus are $\hat{p}$ and $\hat{P}$, respectively, *M* denotes the proton mass, and $m_{e}$ is the mass of the electron.

The time propagation is performed with the split-operator method [41] on a grid in the spatial ranges of − 12 Å ≤R≤ + 12 Å and − 6 Å ≤r≤ + 6 Å, using 512 grid points in each direction and a time step of Δt = 0.0024 fs.

Different initial conditions are employed in solving the time-dependent Schrödinger equation. The initial functions are of the form

(6) $$\begin{array}{c} \Psi \left(r,R,0\right)=N_{0}\;e^{-\beta_{0}{\left(R-R_{0}\right)}^{2}}\;\varphi_{n}\left(r;R\right). \end{array}$$

Here, $N_{0}$ is a normalization constant, and the appearing Gaussian is characterized by its center $R_{0}$ and the width parameter $\beta_{0}$, which is set to a value of 7.14 Å${}^{-2}$ throughout, and the center of the Gaussian $R_{0}$ takes on different values. Solving the electronic Schrödinger equation

(7) $$\begin{array}{c} \left[\frac{{\hat{p}}^{\;2}}{2m_{e}}+V\left(r,R\right)\right]\phi_{n}\left(r;R\right)=V_{n}\left(R\right)\;\phi_{n}\left(r;R\right), \end{array}$$

using imaginary time propagation [42] yields the electronic wave functions $\varphi_{n}\left(r;R\right)$ and the adiabatic potentials $V_{n}\left(R\right)$ corresponding to the electronic state with quantum number (n).

**Figure 1:**
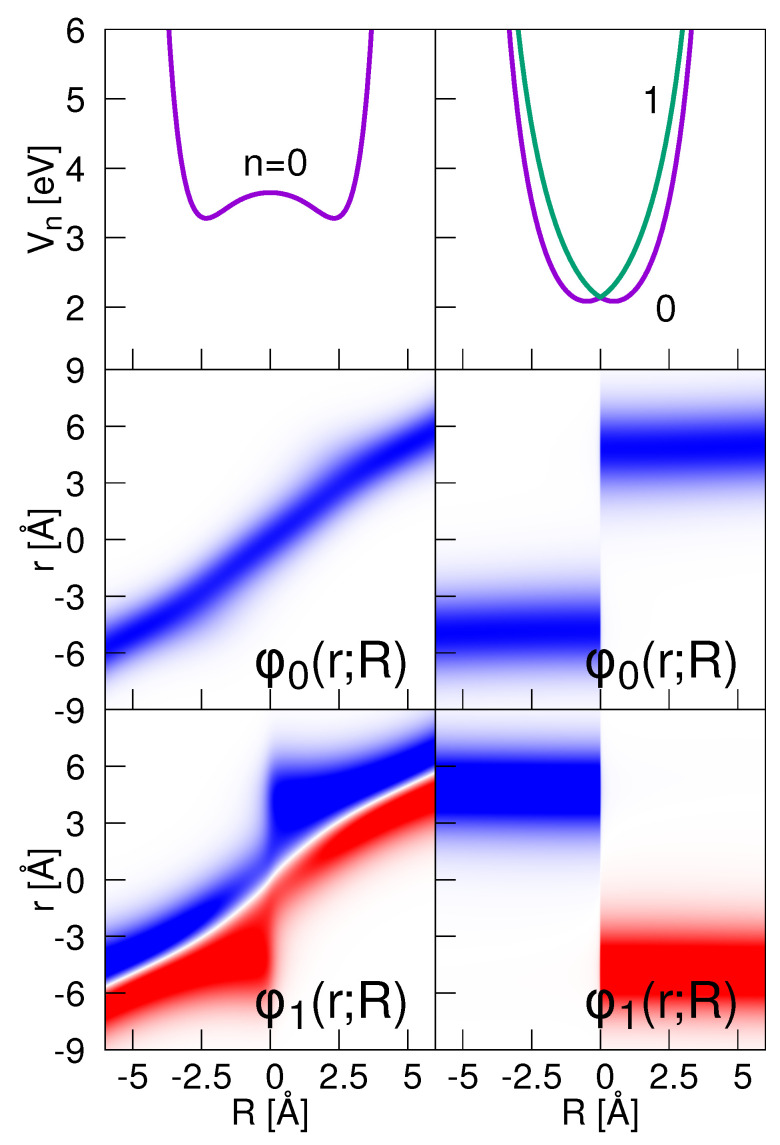
Upper panels: Adiabatic potential curves $V_{n}\left(R\right)$ obtained for two choices of the screening parameter $R_{c}$. The left- and right-hand columns are associated with the cases of a BO motion $(R_{c}=1$ Å) and a diabatic motion $(R_{c}=5$ Å), respectively. The two lower rows show the electronic eigenfunctions $\varphi_{0}\left(r;R\right)$ and $\varphi_{1}\left(r;R\right)$ as indicated.

A variation of the screening parameter entering into the interaction potential leads to different adiabatic potentials. The ground state potential obtained for a value of $R_{c}$ = 1 Å is shown in the left upper panel of Figure 1. The energy gap to the potential $V_{1}\left(R\right)$ is about 4 eV (not shown) so that here, the electronic ground state is separated from the excited electronic states, and we encounter a case where the BO approximation is valid (Section 3.1). The electronic eigenfunctions $\varphi_{0}\left(r;R\right)$ and $\varphi_{1}\left(r;R\right)$ are also contained in the figure (left middle and left lower panel). The ground state function shifts almost linearly with increasing values of the nuclear coordinate, thereby approximately keeping a Gaussian-like shape of constant width. This is not true for $\varphi_{1}\left(r;R\right)$, which varies considerably in its width.

The situation of a strong coupling is illustrated in the right-hand column of Figure 1, and it is obtained in setting $R_{c}$ = 5 Å. The potentials of the two lowest states show a very small energy gap of $[V_{1}\left(0\right)-V_{0}\left(0\right)]<$0.01 eV at the avoided crossing. The respective electronic eigenfunctions, displayed in the lower right two panels of the figure, do not change their form in varying *R* at negative distances. Reaching the avoided crossing at *R* = 0, a sudden jump of the probability density occurs, and then, at positive values of *R*, the shape again remains invariant upon a change in geometry. This is characteristic for the diabatic dynamics, see Section 3.2.

### 2.2. Differential Entropies for Electronic–Nuclear Motion

In order to calculate entropies, according to Equations (1) and (2), probability densities are needed. From the time-dependent wave function, the coordinate probability density is calculated as

(8) $$\begin{array}{c} \rho \left(r,R,t\right)={\left|\Psi \left(r,R,t\right)\right|}^{2}. \end{array}$$

Single-particle densities are obtained by integration. This leads to the electronic density

(9) $$\begin{array}{c} \rho^{el}\left(r,t\right)=\int dR\;\rho \left(r,R,t\right), \end{array}$$

and for the nucleus, one has

(10) $$\begin{array}{c} \rho^{nuc}\left(R,t\right)=\int dr\;\rho \left(r,R,t\right). \end{array}$$

A two-dimensional Fourier transform of Ψ(r,R,t) yields the momentum-space wave function Ψ(p,P,t) and the density

(11) $$\begin{array}{c} \rho \left(p,P,t\right)={\left|\Psi \left(p,P,t\right)\right|}^{2}, \end{array}$$

with *p* and *P* being the electronic and nuclear momenta, respectively. We then have

(12) $$\begin{array}{ccc} \rho^{el}\left(p,t\right) & = & \int dP\;\rho (p,P,t), \end{array}$$

(13) $$\begin{array}{ccc} \rho^{nuc}\left(P,t\right) & = & \int dp\;\rho (p,P,t). \end{array}$$

Using the coordinate- and momentum-space densities, the total differential Shannon entropies can be calculated as follows:

(14) $$\begin{array}{ccc} S_{x}\left(t\right) & = & -\int dr\;\int dR\;\rho \left(r,R,t\right)\;\ln[\rho (r,R,t)], \end{array}$$

(15) $$\begin{array}{ccc} S_{\pi }\left(t\right) & = & -\int dp\;\int dP\;\rho \left(p,P,t\right)\;\ln[\rho (p,P,t)]. \end{array}$$

For the single particles, one obtains the following entropies:

(16) $$\begin{array}{ccc} S_{r}^{el}\left(t\right) & = & -\int dr\;\rho^{el}\left(r,t\right)\;\ln[\rho^{el}\left(r,t\right)], \end{array}$$

(17) $$\begin{array}{ccc} S_{p}^{el}\left(t\right) & = & -\int dp\;\rho^{el}\left(p,t\right)\;\ln[\rho^{el}\left(p,t\right)], \end{array}$$

(18) $$\begin{array}{ccc} S_{R}^{nuc}\left(t\right) & = & -\int dR\;\;\rho^{nuc}\left(R,t\right)\;\ln[\rho^{nuc}\left(R,t\right)], \end{array}$$

(19) $$\begin{array}{ccc} S_{P}^{nuc}\left(t\right) & = & -\int dP\;\;\rho^{nuc}\left(P,t\right)\;\ln[\rho^{nuc}\left(P,t\right)]. \end{array}$$

Another quantity is the “mutual information” (MI). This function contains information on the correlation between different particles [21,31]. In the present situation of a coupled electron–nuclear motion, we derive from the densities the coordinate-space and momentum-space MI:

(20) $$\begin{array}{ccc} I_{x}\left(t\right) & = & S_{r}^{el}\left(t\right)+S_{R}^{nuc}\left(t\right)-S_{x}\left(t\right), \end{array}$$

(21) $$\begin{array}{ccc} I_{\pi }\left(t\right) & = & S_{p}^{el}\left(t\right)+S_{P}^{nuc}\left(t\right)-S_{\pi }\left(t\right). \end{array}$$

We also regard two additional measures for correlation. The first one is the “covariance” [43], which is defined in terms of expectation values of the coordinates *r* and *R* or the momenta *p* and *P* as

(22) $$\begin{array}{ccc} cov_{x}\left(t\right) & = & {\langle rR\rangle }_{t}-{\langle r\rangle }_{t}{\langle R\rangle }_{t}, \end{array}$$

(23) $$\begin{array}{ccc} cov_{\pi }\left(t\right) & = & {\langle pP\rangle }_{t}-{\langle p\rangle }_{t}{\langle P\rangle }_{t}. \end{array}$$

Using the variances for the variables y=r,R,p,P

(24) $$\begin{array}{ccc} \sigma_{y}^{2}\left(t\right) & = & {\langle y^{2}\rangle }_{t}-{\langle y\rangle }_{t}^{2}, \end{array}$$

one defines the “correlations” as

(25) $$\begin{array}{ccc} corr_{x}\left(t\right) & = & \frac{cov_{x}\left(t\right)}{\sigma_{R}\left(t\right)\sigma_{r}\left(t\right)}, \end{array}$$

(26) $$\begin{array}{ccc} corr_{\pi }\left(t\right) & = & \frac{cov_{\pi }\left(t\right)}{\sigma_{P}\left(t\right)\sigma_{p}\left(t\right)}. \end{array}$$

## 3. Results

### 3.1. Weak Coupling: Born–Oppenheimer Dynamics

Setting the screening parameter to a value of $R_{c}$ = 1 Å and imposing the initial condition given in Equation (6) with $R_{0}=-3.5$ Å and with the electronic wave function $\varphi_{0}\left(r;R\right)$ yields the dynamics which exclusively takes place in the electronic ground state. This means that the population ${\tilde{P}}_{0}\left(t\right)={\left|\langle \varphi_{0}|\Psi \left(t\right)\rangle \right|}^{2}$ remains equal to one at all times regarded (where the numerical deviations are in the order of 0.2%).

In a former paper, we used a Gaussian ansatz for the BO wave function to analyze the numerically determined entropies and correlation measures [32]. This function reads

(27) $$\begin{array}{c} \Psi \left(r,R,t\right)=N_{t}\;e^{-\frac{\beta_{t}}{2}{\left(R-R_{t}\right)}^{2}}\;e^{-\frac{\gamma }{2}{\left(r-R\right)}^{2}}, \end{array}$$

where the normalization factor is

(28) $$\begin{array}{c} N_{t}={\left[\frac{\sqrt{\gamma \beta_{t}}}{\pi }\right]}^{\frac{1}{2}}. \end{array}$$

The time dependence of the wave function is contained in the center of the nuclear Gaussian at $R=R_{t}$ and also its width, which is determined by the parameter $\beta_{t}$. Phase factors are thus not included in the ansatz. On the other hand, the electronic wave function is assumed to have a constant width (i.e., γ = const.), and its center shifts linearly with the nuclear coordinate *R*. These assumptions are approximately fulfilled for $\varphi_{0}\left(r;R\right)$, as it can be taken from Figure 1, middle left panel. The ansatz of Equation (27) allows to calculate the various quantities derived from the coordinate-space densities. The details of these calculations can be found in Ref. [44]. Here, we additionally need the respective equations evolving from a momentum-space analysis. The latter is presented in Appendix A. The results for the entropies, variances and correlation measurements are as follows:

(29) $$\begin{array}{ccc} S_{x}\left(t\right) & = & \ln\left[\frac{\pi }{\sqrt{\gamma \beta_{t}}}\right]+1, \end{array}$$

(30) $$\begin{array}{ccc} S_{\pi }\left(t\right) & = & \ln\left[\sqrt{\gamma \beta_{t}}\pi \right]+1, \end{array}$$

(31) $$\begin{array}{ccc} S_{r}^{el}\left(t\right) & = & \ln\left[\sqrt{\frac{(\beta_{t}+\gamma )\pi }{\beta_{t}\gamma }}\;\right]+\frac{1}{2}, \end{array}$$

(32) $$\begin{array}{ccc} S_{p}^{el}\left(t\right) & = & \ln\left[\sqrt{\pi \gamma }\right]+\frac{1}{2}, \end{array}$$

(33) $$\begin{array}{ccc} S_{R}^{nuc}\left(t\right) & = & \ln\left[\sqrt{\frac{\pi }{\beta_{t}}}\right]+\frac{1}{2}, \end{array}$$

(34) $$\begin{array}{ccc} S_{P}^{nuc}\left(t\right) & = & \ln\left[\sqrt{\pi (\beta_{t}+\gamma )}\right]+\frac{1}{2}, \end{array}$$

(35) $$\begin{array}{ccc} I_{x}\left(t\right) & = & \frac{1}{2}\ln\left[1+\frac{\gamma }{\beta_{t}}\right], \end{array}$$

(36) $$\begin{array}{ccc} I_{\pi }\left(t\right) & = & \frac{1}{2}\ln\left[1+\frac{\gamma }{\beta_{t}}\right], \end{array}$$

(37) $$\begin{array}{ccc} \sigma_{r}^{2}\left(t\right) & = & \frac{1}{2}\frac{\gamma +\beta_{t}}{\gamma \beta_{t}}, \end{array}$$

(38) $$\begin{array}{ccc} \sigma_{p}^{2}\left(t\right) & = & \frac{\gamma }{2}, \end{array}$$

(39) $$\begin{array}{ccc} \sigma_{R}^{2}\left(t\right) & = & \frac{1}{2\beta_{t}}, \end{array}$$

(40) $$\begin{array}{ccc} \sigma_{P}^{2}\left(t\right) & = & \frac{1}{2}\left(\beta_{t}+\gamma \right), \end{array}$$

(41) $$\begin{array}{ccc} cov_{x}\left(t\right) & = & \frac{1}{2\beta_{t}}, \end{array}$$

(42) $$\begin{array}{ccc} cov_{\pi }\left(t\right) & = & -\frac{1}{2}\gamma , \end{array}$$

(43) $$\begin{array}{ccc} corr_{x}\left(t\right) & = & \frac{1}{\sqrt{1+\beta_{t}/\gamma }}, \end{array}$$

(44) $$\begin{array}{ccc} corr_{\pi }\left(t\right) & = & -\frac{1}{\sqrt{1+\beta_{t}/\gamma }}. \end{array}$$

In this section, the latter equations are used—as far as possible—for the interpretation of the numerical results.

**Figure 2:**
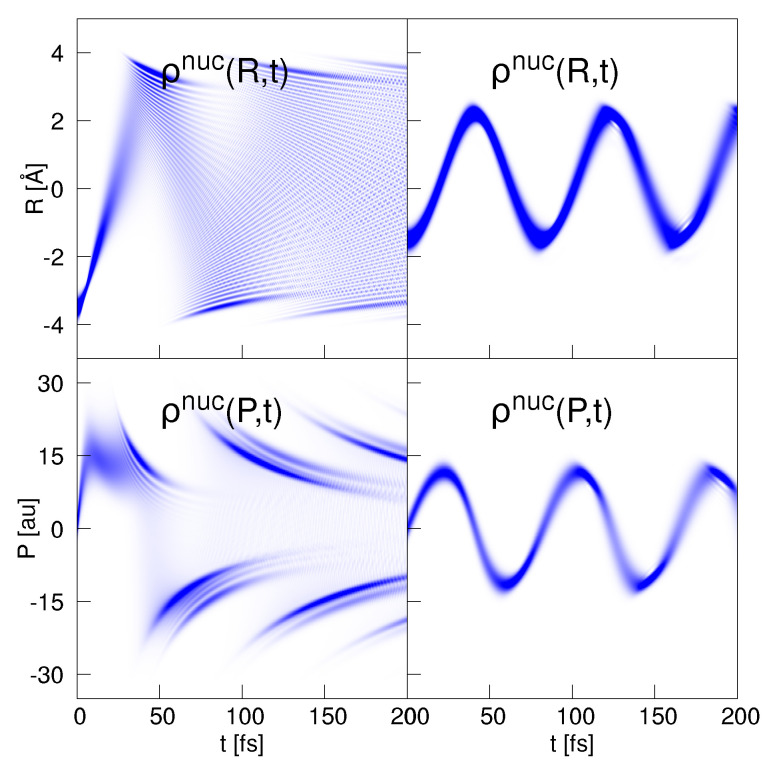
Nuclear density dynamics in the presence of weak (BO dynamics, left-hand column) and strong non-adiabatic coupling (diabatic dynamics, right-hand column). The upper panels show the nuclear densities in coordinate space and the lower panels in momentum space. While in the weakly coupled case the densities disperse quickly, the strongly coupled case shows quasi-harmonic-dynamics.

The nuclear dynamics is illustrated in Figure 2, left upper panel. It is seen that the probability density performs a vibrational motion, but at the end of the displayed time interval, dispersion causes the density to be distributed over the entire classically allowed region. The corresponding momentum density is shown below the coordinate density. It reveals a complex structure which includes, as is also seen in the coordinate-space density, interference fringes. The latter arise when $\rho^{nuc}\left(R,t\right)$ reverses its direction of motion so that $\rho^{nuc}\left(P,t\right)$ changes from a positive to a negative momentum distribution. It is obvious that the ansatz for the nuclear momentum density given in Equation (A15) cannot accurately describe the numerical result shown in the figure. This, in particular, applies to the seen fringes and also to the rapid change from positive to negative momenta. Nevertheless, the derived analytical entropies still prove to be valuable because they are quantities derived from an integration over all momenta.

**Figure 3:**
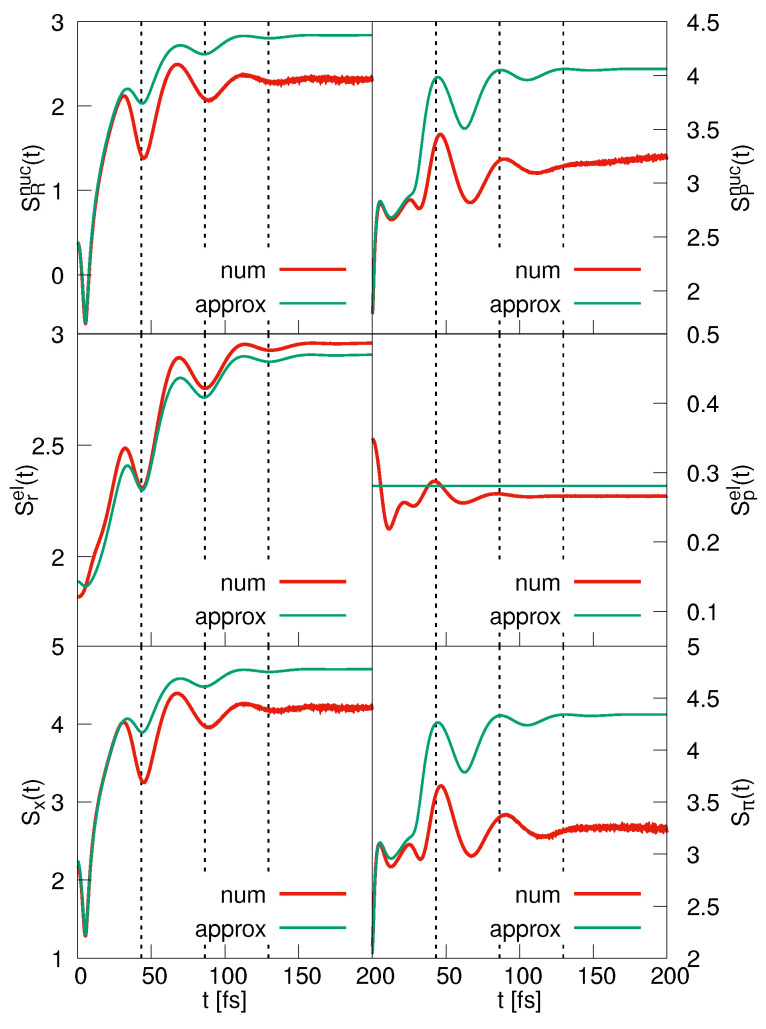
BO dynamics. The left-hand panels show the coordinate-space entropies for the nuclear (**upper panel**) and electronic (**middle panel**) degrees of freedom. Also displayed is the entropy of the coupled system (**lower panel**). The right-hand column contains the same functions derived from the momentum-space densities. In each case, the numerically determined functions are compared to the analytically ones. The dashed lines mark the times when the wave packet reaches the classical turning points of its motion.

The coordinate space nuclear entropy is displayed in the upper left panel of Figure 3. It was checked upon numerically (not shown) that exactly the same curve is obtained if the BO wave function is employed in the calculation. This function is the product of $\varphi_{0}\left(r;R\right)$ and a component $\psi_{0}^{BO}\left(R,t\right)$, which is obtained in solving the nuclear time-dependent Schrödinger equation involving the adiabatic potential $V_{0}\left(R\right)$. We found that the BO approximation is excellent for all entropies presented in the figure. The approximate curve for $S_{R}^{nuc}\left(t\right)$ (Equation (33)) is determined for a value of γ= 0.733 Å, which is calculated in taking an average of the variance $\sigma_{r}^{2}\left(R\right)$ of the electronic eigenfunction in the interval |R|≤ 5 Å. The time-dependent parameter $\beta_{t}$ is obtained from the numerically calculated variance $\sigma_{R}\left(t\right)$ using Equation (39). The analytical expression initially tracks the numerical obtained entropy excellently. Deviations occur when the classical turning point of the wave packet motion is approached for the first time. At this time, the Gaussian approximation to the nuclear density is no longer accurate, see Figure 2. Nevertheless, the analytical curve predicts the time dependence of the nuclear entropy rather well. The minima in the entropy occur at times when the wave packet is focused (large value of $\beta_{t}$) as can be understood from Equation (33). This is the case at the classical turning points of the motion, and it is in accord with the notion that a more localized coordinate space probability density is associated with a larger information on a particle’s position and, in turn, with a smaller entropy. Note that a focusing also takes place around 5 fs, which is due to a squeezing [45] of the wave packet.

The momentum-space nuclear entropy is shown in the right upper panel of Figure 3. To arrive at the approximate entropy, we again use the value of γ = 0.733 Å, and the parameter $\beta_{t}$ is then calculated from the numerically determined variance using Equation (40). The deviations between the numerical and analytical entropies are larger than those found in coordinate space, for the reasons discussed above. Nevertheless, the positions of the extrema are well predicted. From Equation (34), it can be inferred that a minimum is found at times when $\beta_{t}$ assumes a minimum, which correlates with a more localized momentum-space density. Thus, at times when a maximum is found in the coordinate-space entropy, the momentum-space entropy assumes a minimum and vice versa. This illustrates the Fourier relation between the two nuclear densities.

The comparison of Equations (31) and (33) shows that the nuclear and electronic coordinate entropies exhibit minima and maxima at the same times. This indeed is seen if these curves are compared (upper and middle left panel of Figure 3). The agreement between the numerically and analytically determined electronic entropies is astonishingly good. Not unsurprisingly, we find that at the turning points where the electron–nuclear wave packet reverses its motion, we know more precisely where the electron is located as is the case for the nucleus. This is also reflected in the total spatial entropy (lower left panel of the figure). The approximate function $S_{x}\left(t\right)$ is, at all times, larger than the numerical one, which is a property of the normal probability distribution [1].

The electronic momentum-space entropy is contained in the middle right panel of Figure 3. Whereas the analytical solution predicts a time-independent entropy, the numerical results show that there are smaller time variations, where, as for the coordinate-space entropies, the minima and maxima correlate with those found for the nuclear degree of freedom. The time dependence of the total momentum entropy is determined by that of the nuclear entropy because the latter has a more pronounced time dependence as $S_{x}^{el}\left(t\right)$. Note, however, that it is not the sum of the two particle entropies; see the discussion below.

The results presented so far show that the time dependence of the entropies is determined by the nuclear component of the wave function. This, of course, does not come as a surprise because in the present case, the BO approximation is valid, and thus the electronic part of the wave functions does not include time as a parameter. The predictions derived from our Gaussian ansatz for the wave function, namely that the minima in the entropies correlate with a more localized nuclear coordinate-space density, are confirmed by the numerical calculation. Concerning the information available, it is seen that the coordinate-space and momentum-space entropies reflect the Fourier relation between the two spaces. In particular, if we know more about the localization of one or the other particle, less is known about its momentum and vice versa.

**Figure 4:**
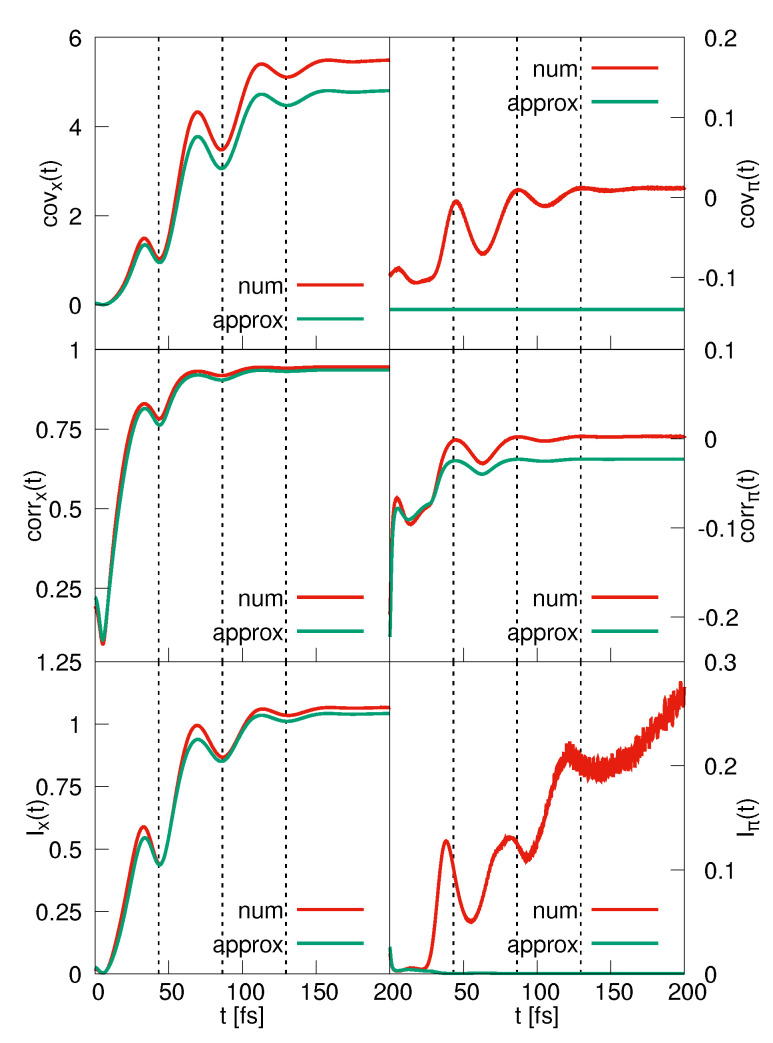
BO-dynamics. The left-hand column shows the coordinate-space covariance, correlation and mutual information, as indicated. The respective curves obtained from the momentum-space densities are depicted in the right-hand column. In each case, numerically and analytically derived results are compared. The times when the turning points are reached are marked by the vertical lines.

Let us, in what follows, discuss the three measures of particle entanglement, namely the covariance, correlation and mutual information as defined in Section 2.2. The covariance functions $cov_{x}\left(t\right)$ are shown in the upper left panel of Figure 4, and it is seen that the analytically derived curve again provides a very good approximation of the numerically exact one. Thus, using Equation (41) for interpretation, a localized nuclear density, corresponding to a large value of $\beta_{t}$, goes in hand with a low degree of particle correlation. The reason is that in this case, the *R*-dependence of the electronic wave function entering into the BO wave function is of minor importance so that the wave function is approximately separable. This shows that at times when the classical turning points (which are indicated as dashed vertical lines in Figure 4) are reached, the covariance takes on minimal values. The same applies to the correlation (middle left panel of Figure 4) and also to the MI (lower left panel). All three functions exhibit a comparable time dependence so that we conclude that they measure the correlation in a very similar way.

A different picture evolves from the momentum-space functions displayed in the right-hand column of Figure 4. The nuclear momentum covariance and correlation behave rather similarly as a function of time. They exhibit an overall decrease, which is modulated with the vibrational period, and for longer times, they level to a value of about zero. Both numerically determined functions are negative initially, and they switch sign for later times, whereas the analytical predictions stay negative throughout. This deviation is a non-BO effect, which was checked upon in performing a BO propagation.

It is seen that at times when the turning points are reached, the momentum correlation approaches zero, which agrees with the behavior in configuration space. Whereas no time dependence appears in the analytical covariance expression, the change of the nuclear variance causes the correlation $corr_{\pi }\left(t\right)$ to vary, similar to the numerical curve.

The momentum-space MI, displayed in the lower right panel of Figure 4, exhibits an unusual behavior. The overall rise of the function is modulated by the vibrational period of the quantum motion. The analytically determined MI does only give reasonable results at very early times. It is interesting to observe that around the times when $corr_{x}\left(t\right)$ and $cov_{x}\left(t\right)$ predict a low degree of particle correlation, the MI does not, i.e., the MI is phase shifted with respect to the other two functions. We also note that the fast oscillations seen in the MI are neither of numerical origin nor are they due to non-BO effects as seen, for example, in time-dependent electron momentum expectation values [46].

**Figure 5:**
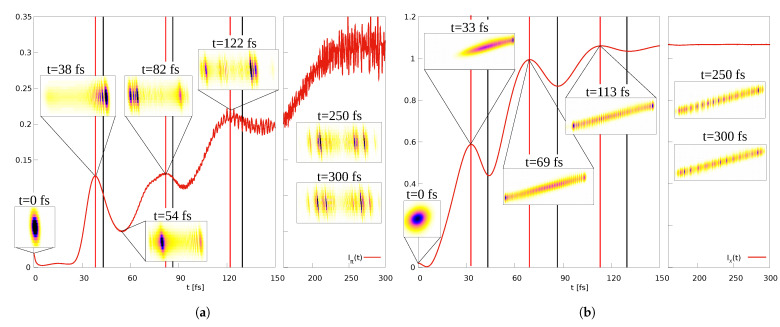
BO-dynamics. (**a**) Shown are momentum-space densities ρ(p,P,t) for different times as indicated. Abscissa and ordinate correspond to the nuclear and electronic momentum, respectively. Also shown is the MI $I_{\pi }\left(t\right)$. The vertical red lines mark times when the MI exhibits extrema, and the black lines indicate the times when the classical turning points are reached. (**b**) Same as (a), but in coordinate space. The densities ρ(r,R,t) (abscissa *R*, ordinate *r*) are depicted for selected times.

To gain more insight into the behavior of $I_{\pi }\left(t\right)$, we show in Figure 5a snapshots of the momentum-space density at times when maxima and minima in the MI occur. It is seen that initially, when $I_{\pi }\left(t\right)$ is small, the density is a nodeless Gaussian-like distribution. At a time of *t* = 38 fs, there appears a clear nodal structure, and the MI takes on a maximum. Then, at 54 fs, the density in the region of its largest amplitude has lost the nodal pattern (although, at larger nuclear momenta there is a region where the density shows nodes, but the overall amplitude is small). Nodes appear another time at the location of the next maximum (82 fs). A similar trend is seen at later times. The conclusion is that an increase in the MI goes in hand with the appearance of nodal patterns in the momentum-space density found in the direction of the nuclear momentum, and that with an increasing number of nodes, the MI grows. To observe this behavior, it is important that the nodes are not oriented parallel to the axes because otherwise they do not give a contribution to wave packet entanglement. This is the case for the coordinate-space density. The latter is depicted, for selected times, in Figure 5b. Starting with a Gaussian-like function at time zero, the density moves along the line r=R. There also appears a nodal structure but here, all nodal lines are oriented perpendicular to the nuclear coordinate axis. This does not change as a function of time as is illustrated for the times *t* = 69 fs and 113 fs, where the coordinate-space MI assumes maxima. At later times (250 fs and 300 fs), the densities are quite similar, which leads to a constant value of $I_{x}\left(t\right)$. A clearer nodal pattern is seen in the momentum-space density, which, for these times, fluctuates as a function of time (Figure 5a), giving rise to fluctuations in $I_{\pi }\left(t\right)$.

To support the connection between the nodal structure of the momentum-space density and the resulting MI, we developed an analytical model using the simple ansatz for the normalized density as

(45) $$\begin{array}{c} \rho_{a,b}\left(p,P\right)=\frac{2\;e^{-P^{2}-p^{2}}\cos^{2}\left(\frac{a}{\sqrt{1+b^{2}}}\left(P+bp\right)\right)}{\pi (1+e^{-a^{2}})}\;. \end{array}$$

Here, the parameter *a* determines the frequency of the cosine and thus the number of nodes. In the limit a→0, a standard non-correlated Gaussian is recovered. The second parameter b∈[0,1] determines the alignment of the nodes. The factor $\sqrt{1+b^{2}}$ ensures that *b* does not distort the density and thus influences the effective frequency/number of nodes.

Using *MATHEMATICA*, we calculate the MI as a function of the frequency (i.e., number of nodes) and for several values of *b*. The extensive analysis of these calculations is out of the scope of this paper, and it will be given elsewhere [44]. Here, we summarize the main results. Regarding the MI as a function of the number of nodes, we find that it grows monotonically and approaches a constant with increasing number of nodes. Furthermore, the MI vanishes for b=0 and arbitrary *a*, and curves reach the same upper bound faster with increasing b>0. The rough model explains qualitatively what is seen in the numerical results. Using the ansatz Equation (45), the covariance and correlation can be calculated analytically. These functions vanish for b→0 and a→0 as expected, but they also vanish for a→∞, whereas the MI approaches a finite non-zero limit. This finding is also in accord with what is seen in Figure 4. Here we encounter a behavior of the MI which is different from the covariance and correlation, which hints at the fact that non-linear correlations are present in the wave packet moving in momentum space.

### 3.2. Strong Coupling: Diabatic Dynamics

In this section, we treat the case of a strong non-adiabatic coupling, which is achieved in setting the screening parameter to $R_{c}$ = 5 Å. The adiabatic potentials and the electronic eigenfunctions are illustrated in Figure 1. The nuclear wave function starts at $R_{0}$ = −1.5 Å. For an analytical approach, we take advantage of the fact that the dynamics (see right-hand column of Figure 2) can be well described as a diabatic motion [47], where the wave function is of the form

(46) $$\begin{array}{c} \Psi \left(r,R,t\right)=\psi_{d}\left(R,t\right)\;\varphi_{0}\left(r,R_{d}\right). \end{array}$$

Within this approximation, the nuclear component moves in the diabatic potential obtained in connecting the negative branch of $V_{0}\left(R\right)$ with the positive branch of $V_{1}\left(R\right)$, and the electronic wave function is the diabatic function calculated at a fixed value $R=R_{d}$.

The analytical treatment starts from the ansatz for the wave function as

(47) $$\begin{array}{c} \Psi \left(r,R,t\right)=\sqrt{\frac{\sqrt{\gamma \beta_{t}}}{\pi }}\;e^{-\frac{\beta_{t}}{2}{\left(R-R_{t}\right)}^{2}}\;e^{-\frac{\gamma }{2}{\left(r-R_{d}\right)}^{2}}. \end{array}$$

Thus, here, the total wave function is separable, which simplifies the calculations if compared to the BO case treated in Section 3.1. As shown in Appendix B, the following entropies evolve from the diabatic ansatz of the wave function:

(48) $$\begin{array}{ccc} S_{x}\left(t\right) & = & \ln\left[\frac{\pi }{\sqrt{\beta_{t}\gamma }}\right]+1, \end{array}$$

(49) $$\begin{array}{ccc} S_{\pi }\left(t\right) & = & \ln\left[\sqrt{\gamma \beta_{t}}\pi \right]+1, \end{array}$$

(50) $$\begin{array}{ccc} S_{r}^{el}\left(t\right) & = & \ln\left[\sqrt{\frac{\pi }{\gamma }}\right]+\frac{1}{2}, \end{array}$$

(51) $$\begin{array}{ccc} S_{R}^{nuc}\left(t\right) & = & \ln\left[\sqrt{\frac{\pi }{\beta_{t}}}\right]+\frac{1}{2}, \end{array}$$

(52) $$\begin{array}{ccc} S_{p}^{el}\left(t\right) & = & \ln\left[\sqrt{\pi \gamma }\right]+\frac{1}{2}, \end{array}$$

(53) $$\begin{array}{ccc} S_{P}^{nuc}\left(t\right) & = & \ln\left[\sqrt{\pi \beta_{t}}\right]+\frac{1}{2}. \end{array}$$

Here, we determine γ from the electronic width at t=0 which yields a value of γ=0.436 Å${}^{-2}$. Note that because of the separability of the diabatic wave function, the correlation, covariance and mutual information vanish in coordinate- and momentum space.

A comparison of the equations for the coordinate and momentum total entropies in the diabatic and adiabatic case (Equations (29) and (48), Equations (30) and (49)) shows that they are identical. In both situations, the nuclear dynamics takes place in a single potential. For a diabatic motion, the electron remains stationary, whereas the nucleus vibrates, being more or less localized as time goes along. In the BO case, both particles localize simultaneously.

**Figure 6:**
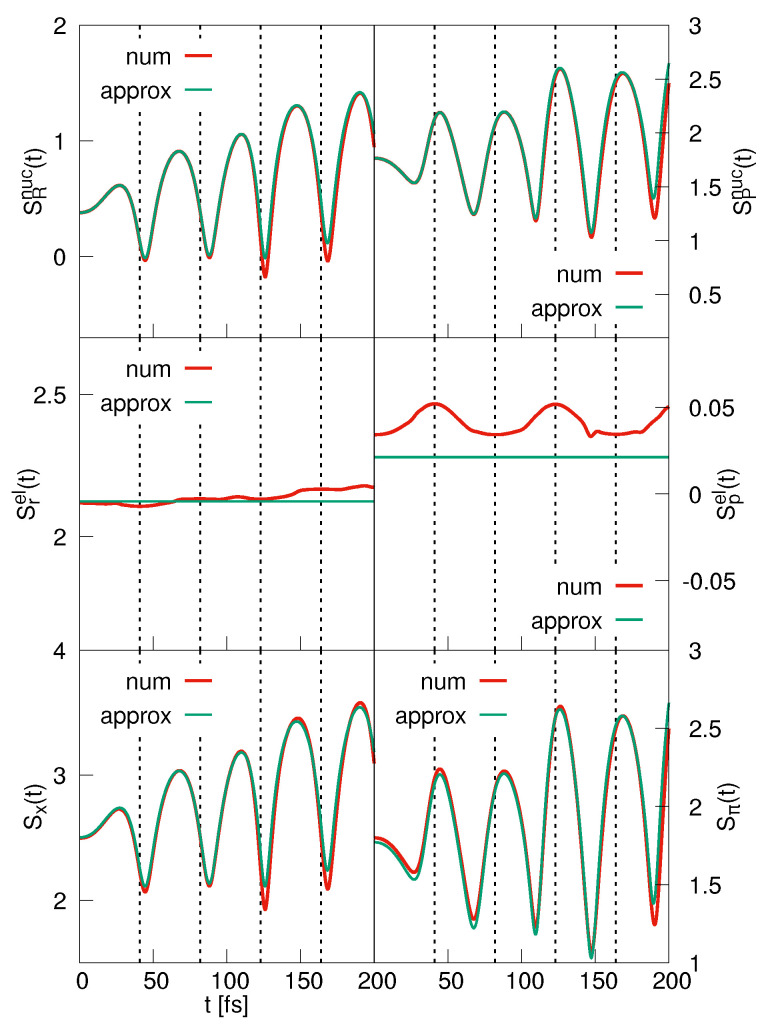
Same as Figure 3 but for the strong coupling case.

The entropies evolving from the diabatic dynamics are presented in Figure 6. The nuclear coordinate entropy oscillates with a single frequency associated with the vibrational wave packet motion, and the analytically obtained curve tracks the numerical one perfectly. The oscillations show an increasing amplitude, which, according to Equation (51)), correlates with a decrease in the width parameter $\beta_{t}$. The obtained curve is much more regular if compared to the BO case (Figure 3). This is due to the excellent accuracy of the diabatic approximation. Here, the nuclear motion takes place in an almost harmonic potential (which is not the case in the adiabatic situation, where the potential shows a double minimum structure). This harmonic-like motion is clearly seen in the density dynamics displayed in the right-hand column of Figure 2. The momentum space nuclear entropy $S_{P}^{nuc}\left(t\right)$ exhibits the same quasi-periodic time structure but is phase shifted with respect to $S_{R}^{nuc}\left(t\right)$, as is expected from Equation (53). In both spaces, the electronic entropy is nearly constant as is predicted within the analytical ansatz (Equations (50) and (52)). The minor numerically found deviations from a constant behavior are due to the approximate nature of the diabatic ansatz and also the variation of the electronic variance as a function of the nuclear coordinate s*R*.

Whereas for the purely diabatic dynamics, all functions which measure correlations are identical to zero, the numerically calculated curves are non-zero but are small in magnitude, and thus we do not show them here.

Until now, we adopted a diabatic picture to describe the strong coupling case. It is interesting to relate the results to the adiabatic approach, where the expansion of the total wave function reads

(54) $$\begin{array}{c} \Psi \left(r,R,t\right)=\sum_{n=0}^{\infty }\psi_{n}\left(R,t\right)\;\varphi_{n}\left(r;R\right). \end{array}$$

The total probability density then is

(55) $$\begin{array}{c} \rho \left(r,R,t\right)={\left|\Psi \left(r,R,t\right)\right|}^{2}=\sum_{n,m=0}^{\infty }\rho_{nm}\left(r,R,t\right), \end{array}$$

with the matrix elements

(56) $$\begin{array}{c} \rho_{nm}\left(r,R,t\right)=\psi_{n}^{*}\left(R,t\right)\;\psi_{m}\left(R\right)\;\phi_{n}\left(r;R\right)\;\phi_{m}\left(r;R\right). \end{array}$$

In our example, we start in the first excited electronic state, and the dynamics then leads to an almost 100% population transfer to the ground state at a time of $t_{tr}$ ≈ 18.8 fs. Later on, the population exchange between the two states occurs periodically. In order to illustrate the contributions of the different states to the entropies, we decompose the latter into components. Therefore, we first calculate adiabatic nuclear densities by integration as

(57) $$\begin{array}{ccc} \rho_{nm}^{nuc}\left(R,t\right) & = & \int dr\;\rho_{nm}\left(r,R,t\right)={\left|\psi_{n}\left(R,t\right)\right|}^{2}\;\delta_{nm}. \end{array}$$

Thus, the off-diagonal elements vanish. This, in general, does not apply to the electronic case where we have

(58) $$\begin{array}{ccc} \rho_{nm}^{el}\left(r,t\right) & = & \int dR\;\rho_{nm}\left(r,R,t\right). \end{array}$$

In the present numerical example, we find that the off-diagonal elements are negligible. The diagonal terms are positive semi-definite and may be interpreted as densities which are not normalized, and they are related to the populations in the electronic states as

(59) $$\begin{array}{c} {\tilde{P}}_{n}\left(t\right)={\left|\langle \varphi_{n}|\Psi \left(t\right)\rangle \right|}^{2}=\int dr\int dR\;\rho_{nn}\left(r,R,t\right). \end{array}$$

Using the diagonal elements of the densities, we define state-specific entropies as follows:

(60) $$\begin{array}{ccc} S_{x,n}\left(t\right) & = & -\int dR\int dr\;\rho_{nn}\left(r,R,t\right)\;\ln\left[\rho_{nn}\left(r,R,t\right)\right], \end{array}$$

(61) $$\begin{array}{ccc} S_{r,n}^{el}\left(t\right) & = & -\int dr\;\rho_{nn}^{el}\left(r,t\right)\;\ln\left[\rho_{nn}\left(r,t\right)\right], \end{array}$$

(62) $$\begin{array}{ccc} S_{R,n}^{nuc}\left(t\right) & = & -\int dR\;\rho_{nn}^{nuc}\left(R,t\right)\;\ln\left[\rho_{nn}\left(R,t\right)\right]. \end{array}$$

The decomposition of the entropies into different components may as well be performed in momentum space. Taking the Fourier transform of the wave function yields

(63) $$\begin{array}{ccc} \Psi (p,P,t) & = & \sum_{n}\Psi_{n}\left(p,P,t\right), \end{array}$$

with the definition

(64) $$\begin{array}{ccc} \Psi_{n}\left(r,P,t\right) & = & \frac{1}{2\pi }\int dR\;e^{-iPR}\psi_{n}\left(R,t\right)\int dr\;e^{-ipr}\varphi_{n}\left(r;R\right). \end{array}$$

The decomposition of the momentum-space densities is calculated as

(65) $$\begin{array}{c} \rho \left(p,P,t\right)=\sum_{n,m}\rho_{nm}\left(p,P,t\right), \end{array}$$

with

(66) $$\begin{array}{c} \rho_{nm}\left(p,P,t\right)=\Psi_{n}^{*}\left(p,P,t\right)\;\psi_{m}\left(p,P,t\right). \end{array}$$

From the latter matrix elements, we derive the electronic and nuclear matrix elements

(67) $$\begin{array}{ccc} \rho_{nm}^{el}\left(p,t\right) & = & \int dP\;\rho_{nm}\left(p,P,t\right), \end{array}$$

(68) $$\begin{array}{ccc} \rho_{nm}^{nuc}\left(P,t\right) & = & \int dp\;\rho_{nm}\left(p,P,t\right). \end{array}$$

The state-specific entropies are defined incorporating the diagonal elements of the densities and read

(69) $$\begin{array}{ccc} S_{\pi ,n}\left(t\right) & = & -\int dP\int dp\;\rho_{nn}\left(p,P,t\right)\;\ln\left[\rho_{nn}\left(p,P,t\right)\right], \end{array}$$

(70) $$\begin{array}{ccc} S_{p,n}^{el}\left(t\right) & = & -\int dp\;\rho_{nn}^{el}\left(p,t\right)\;\ln\left[\rho_{nn}^{el}\left(p,t\right)\right], \end{array}$$

(71) $$\begin{array}{ccc} S_{P,n}^{nuc}\left(t\right) & = & -\int dP\;\rho_{nn}^{nuc}\left(P,t\right)\;\ln\left[\rho_{nn}^{nuc}\left(P,t\right)\right]. \end{array}$$

**Figure 7:**
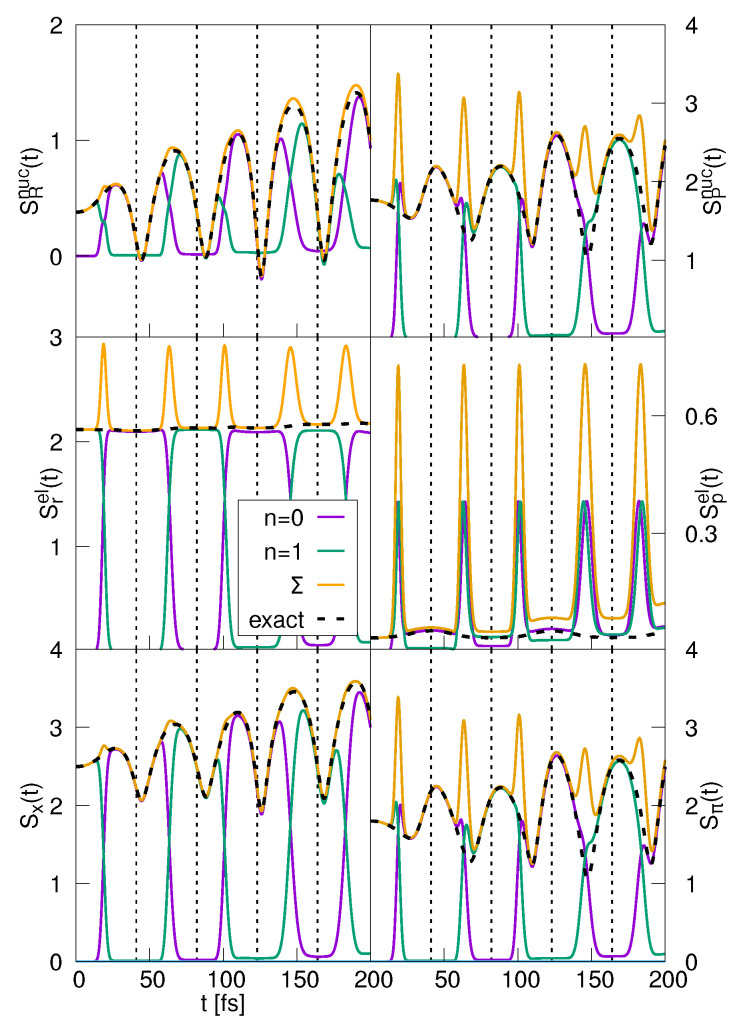
Decomposition of the entropies in the strong coupling case: Nuclear (**upper panels**), electronic (**middle panels**) and total entropies (**lower panels**). The coordinate-space entropies ($S_{R}^{nuc}\left(t\right),S_{r}^{el}\left(t\right),S_{x}\left(t\right)$) and the contributions of the two adiabatic electronic states ($S_{R,n}^{nuc}\left(t\right),S_{r,n}^{el}\left(t\right),S_{x,n}\left(t\right),n=0,1$) are shown, and also the sum (Σ) of the state-specific entropies and the numerically exact curve. The right-hand column contains the respective quantities derived from the momentum-space density. The vertical dashed lines indicate the times when the classical turning points are reached.

In the left-hand column of Figure 7, we show the results of the decomposition of the coordinate-space entropies. The nuclear functions $S_{R,n}\left(t\right)$ for the quantum numbers n=0,1 are displayed in the upper left-hand panel of the figure. Also included is the sum of these two components and the numerically determined nuclear entropy $S_{R}\left(t\right)$. The components follow the population dynamics and also reflect the focusing and broadening of the wave packet components in the two states. The term $S_{R,1}\left(t\right)$ contributes to the entropy until the non-adiabatic transition takes place. Then, this function decreases, which goes in hand with an increase in $S_{R,0}\left(t\right)$ until the latter function becomes equal to the nuclear entropy. This behavior takes place several times in the shown interval, and, besides minor deviations, at all times, the sum of the two components equals the numerically determined nuclear entropy. To understand this, we write the latter within the approximation of two contributing states:

(72) $$\begin{array}{ccc} S_{R}^{nuc}\left(t\right)= & - & \int dR\;|\psi_{0}{\left(R,t\right)|}^{2}\ln\left[|\psi_{0}{\left(R,t\right)|}^{2}+{\left|\psi_{1}\left(R,t\right)\right|}^{2}\right]\\ & - & \int dR\;|\psi_{1}{\left(R,t\right)|}^{2}\ln\left[|\psi_{0}{\left(R,t\right)|}^{2}+{\left|\psi_{1}\left(R,t\right)\right|}^{2}\right]. \end{array}$$

From our numerical calculation, we find that the two nuclear wave functions $\psi_{0}\left(R,t\right)$ and $\psi_{1}\left(R,t\right)$ at no time have a significant spatial overlap, even around the transition times. Thus, we may set $\psi_{1}\left(R,t\right)$ equal to zero in the first integral appearing in Equation (72) and neglect $\psi_{0}\left(R,t\right)$ in the second integral. This yields

(73) $$\begin{array}{ccc} S_{R}^{nuc}\left(t\right) & \approx & -\sum_{n=0,1}\int dR\;{\left|\psi_{n}\left(R,t\right)\right|}^{2}\ln\left[|\psi_{n}{\left(R,t\right)|}^{2}\right]=\sum_{n=0,1}S_{R,n}^{nuc}\left(t\right). \end{array}$$

The electronic state-specific entropies (middle left panel of Figure 7) behave like the populations in the two electronic states (the curves for ${\tilde{P}}_{n}\left(t\right)$ are not included because their time variation is almost identical to the functions $S_{R,n}\left(t\right)$). This does not apply in the time intervals where the transitions take place. There, the sum of the electronic components $S_{r,n}^{el}\left(t\right)$ does not add up to the electronic coordinate entropy. Because the off-diagonal elements of the matrix $\rho_{nm}\left(r,t\right)$ are negligible, we have

(74) $$\begin{array}{c} S_{r}^{el}\left(t\right)=-\int dr\left[\sum_{n=0}^{1}\rho_{nn}^{el}\left(r,t\right)\right]\ln\left[\sum_{n=0}^{1}\rho_{nn}^{el}\left(r,t\right)\right]. \end{array}$$

Numerically, we find that at a transition time $t_{tr}$, the elements $\rho_{00}^{el}\left(r,t\right)$ and $\rho_{11}^{el}\left(r,t\right)$ are approximately equal. It then follows that

(75) $$\begin{array}{ccc} S_{r}^{el}\left(t_{tr}\right) & \approx & -\int dr[2\rho_{00}^{el}\left(r,t_{tr}\right)]\ln[2\rho_{00}^{el}\left(r,t_{tr}\right)]\\ & = & -\ln\left[2\right]\int dr[2\rho_{00}^{el}\left(r,t_{tr}\right)]-2\int dr\rho_{00}^{el}\left(r,t_{tr}\right)\ln[\rho_{00}^{el}\left(r,t_{tr}\right)]\\ & = & -\ln\left[2\right]+S_{0,r}\left(t_{tr}\right)+S_{1,r}\left(t_{tr}\right), \end{array}$$

where we used that at the transition time, the population takes a value of ${\tilde{P}}_{0}\left(t_{tr}\right)$ = 0.5. Thus, at this time, the numerical electronic entropy and the sum of the components differ by a value of −ln[2]. This is in accord with what is seen in Figure 7. In contrast, the total entropy is excellently represented by the sum of the state functions, which can be traced back to the fact that the nuclear components of the two involved states do not overlap and thus, as a result of integrating out the nuclear degree of freedom, the same applies to the diagonal elements $\rho_{nn}$.

In the right-hand column of Figure 7, we document the decomposition of the momentum-space entropies. Again, the nuclear state-selective entropies follow the population dynamics, but here the sum of the single components exhibits larger deviations from the total entropy around the transition times. This is because the mathematical structure of the momentum-space matrix elements (Equation (66)) is more complex than in coordinate space. Pronounced deviations are also found in the electronic momentum entropies, where large maxima are seen in the state-specific entropies, whereas the total electronic entropy remains nearly constant. The disagreement of the curves, related to the non-adiabatic transitions, is also apparent in the total momentum entropy, see lower right panel of the figure. This is because the diagonal elements $\rho_{nn}$ overlap already in (p−P)-space, and thus, the off-diagonal elements cannot be ignored.

## 4. Summary

We study differential entropies evolving from a coupled electron–nuclear quantum dynamics. Using the total density and single-particle densities, we calculate the respective time-dependent entropies in coordinate- and momentum space. In doing so, two situations are regarded. In the first one, the dynamics takes place in a single adiabatic electronic state so that the Born–Oppenheimer approximation applies. The second case is characterized by a strong non-adiabatic coupling, which leads to a complete population transfer between two adiabatic states. Under these conditions, one encounters a diabatic motion.

The two described dynamical situations are realized within a model for a one-dimensional motion of a single electron and nucleus, which allows to integrate the time-dependent Schrödinger equation numerically. In both cases, it is also possible to find an analytical description which explains most of the features found in the numerical calculation.

For the BO dynamics, where the electron adiabatically follows the nucleus, the time-dependence of the coordinate-space entropies is determined by the position and width of the nuclear density. In our example, the latter performs a vibrational motion, and at the classical turning points of this dynamics, the density is focused. Then, we have more information about the positions of the two particles, which is reflected in the particle and total entropies, which all pass through minima. At the same instants of time, the momentum-space entropies exhibit maxima which are related to the Fourier properties of the coordinate- and momentum-space wave functions.

We compare three different measures for the particle entanglement, i.e., the covariance, the correlation and the mutual information. If these functions are determined from the coordinate space densities, they show a similar time dependence. In particular, when the wave packet reaches the classical turning point, they exhibit minima. This means that at these times, when the coordinate space wave function becomes more localized, the electron–nuclear correlation is small. The reason is that then the dependence of the electronic wave function on the nuclear geometry is less pronounced. A different picture evolves from the momentum-space densities. There, the MI behaves differently than the covariance and correlation. Because the latter monitor a linear particle entanglement, this hints at nonlinear effects. It is found that the behavior of the MI is related to the nodal structure of the momentum-space density. Maxima occur at times when the latter density exhibits a clear node behavior with lines oriented non-parallel to the nuclear momentum axis. An analytical model shows that with an increasing number of nodes, the MI grows until a threshold is approached.

In the situation where strong non-adiabatic couplings are present, the dynamics is most efficiently described within a diabatic picture, which means that there is no correlation between the particles present. This is clear from the form of the wave function and is seen in our numerical example. The numerical results can be well reproduced using the analytical model starting from a diabatic wave function. The nuclear wave packet motion proceeds in an almost harmonic potential, which results in a regular variation of the nuclear entropy. On the other hand, the electronic entropy is nearly constant. This holds in both coordinate- and momentum space. A decomposition of the coordinate space in terms of the adiabatic expansion of the total wave function leads to state-specific entropies. For the nuclear case, these functions add up to the total entropy. This, however, is not the case in momentum space. There, the decomposition yields non-negligible off-diagonal contributions, which cannot be ignored. 

## Data Availability

The data that support the findings of this study are available from the corresponding author upon reasonable request.

## Abbreviations

The following abbreviations are used in this manuscript:

BO	Born–Oppenheimer
MI	Mutual information

## Appendix A. Entropies in Momentum Space

To evaluate the momentum-space densities, we first take the Fourier transform of the spatial wave function Equation (27):

(A1) $$\begin{array}{ccc} \Psi (p,P) & = & \frac{N_{t}}{2\pi }\int dR\;\int dr\;e^{-iPR-ipr}e^{-\frac{\beta_{t}}{2}{\left(R-R_{t}\right)}^{2}-\frac{\gamma }{2}{\left(r-R\right)}^{2}}. \end{array}$$

The Fourier integral over the electronic coordinate is a Gaussian integration. Using the coordinate $r^{\prime }=r-R$, one finds

(A2) $$\begin{array}{cc} e^{-ipR}\int dr^{\prime }\;e^{-ipr^{\prime }}e^{-\frac{\gamma }{2}r^{\prime 2}}= & =\sqrt{\frac{2\pi }{\gamma }}\;e^{-ipR-\frac{p^{2}}{2\gamma }}. \end{array}$$

With this result, and performing another Gaussian integral, the wave function is evaluated as

(A3) $$\begin{array}{ccc} \Psi (p,P) & = & \frac{N_{t}}{2\pi }\sqrt{\frac{2\pi }{\gamma }}e^{-\frac{\beta_{t}}{2}R_{t}^{2}-\frac{p^{2}}{2\gamma }}\int dR\;e^{-\frac{\beta_{t}}{2}R^{2}+\left[\beta_{t}R_{t}-i\left(p+P\right)\right]R}\\ & = & \frac{N_{t}}{2\pi }\sqrt{\frac{2\pi }{\gamma }}\sqrt{\frac{2\pi }{\beta_{t}}}e^{-\frac{\beta_{t}}{2}R_{t}^{2}-\frac{p^{2}}{2\gamma }}\;e^{\frac{{\left[\beta_{t}R_{t}-i\left(p+P\right)\right]}^{2}}{2\beta }}\\ & = & \frac{1}{\sqrt{\pi }{\left(\beta_{t}\gamma \right)}^{1/4}}\;e^{-\frac{\beta_{t}}{2}R_{t}^{2}-\frac{p^{2}}{2\gamma }+\frac{{\left[\beta_{t}R_{t}-i\left(p+P\right)\right]}^{2}}{2\beta_{t}}}. \end{array}$$

In calculating the momentum-space density, the exponential containing the mean position $R_{t}$ drops out and one finds

(A4) $$\begin{array}{c} \rho \left(p,P,t\right)=M_{t}\;e^{-\frac{1}{\beta_{t}}{\left(p+P\right)}^{2}-\frac{p^{2}}{\gamma }}, \end{array}$$

with the normalization constant

(A5) $$\begin{array}{c} M_{t}=\frac{1}{\pi \sqrt{\beta_{t}\gamma }}. \end{array}$$

To evaluate the entropy, we take advantage of the fact that the polynomial appearing in the exponent is quadratic. Thus, the function

(A6) $$\begin{array}{c} f\left(p,P\right)=-\frac{1}{\beta_{t}}{\left(p+P\right)}^{2}-\frac{p^{2}}{\gamma }, \end{array}$$

using the real valued parameter λ may be written as

(A7) $$\begin{array}{c} f\left(p^{\prime },P^{\prime }\right)=\lambda f\left(p,P\right),\;\;\;p^{\prime }=\sqrt{\lambda }\;p,\;P^{\prime }=\sqrt{\lambda }\;P, \end{array}$$

with the scaled coordinates $p^{\prime }$ and $P^{\prime }$. We then have the property

(A8) $$\begin{array}{c} M_{t}\int dp\int dP\;e^{\lambda f(p,P)}=\frac{1}{\lambda }\;M_{t}\;\int dp^{\prime }\int dP^{\prime }\;e^{f(p^{\prime },P^{\prime })}=\frac{1}{\lambda }, \end{array}$$

and it follows that

(A9) $$\begin{array}{ccc} M_{t}\int dp\int dP\;e^{f(p,P)}\;f\left(p,P\right) & = & \lim_{\lambda \to 1}\frac{d}{d\lambda }\left[M_{t}\int dp\int dP\;e^{\lambda f(p,P)}\right]\\ & = & \lim_{\lambda \to 1}\frac{d}{d\lambda }\left[\frac{1}{\lambda }\right]=-1. \end{array}$$

We note that this result can be generalized to quadratic functions $f(y_{1},y_{2},\ldots ,y_{d})$ depending on *d* variables $y_{j}$. Below, we need the derived property for the case of a single variable *y* so that

(A10) $$\begin{array}{c} M_{t}\int dy\;e^{f(y)}\;f\left(y\right)=\lim_{\lambda \to 1}\frac{d}{d\lambda }\left[M_{t}\int dy\;e^{\lambda f(y)}\right]=\lim_{\lambda \to 1}\frac{d}{d\lambda }\left[\frac{1}{\sqrt{\lambda }}\right]=-\frac{1}{2}. \end{array}$$

The entropy now can be calculated as

(A11) $$\begin{array}{ccc} S_{\pi }\left(t\right) & = & -\ln\left[M_{t}\right]-M_{t}\int dp\int dP\;e^{f(p,P)}\;f\left(p,P\right)=\ln\left[\sqrt{\gamma \beta_{t}}\pi \right]+1. \end{array}$$

For the sum of the momentum and position entropy, one finds

(A12) $$\begin{array}{ccc} S_{\pi }\left(t\right)+S_{x}\left(t\right) & = & \ln\left[\sqrt{\gamma \beta_{t}}\pi \right]+1+\ln\left[\frac{\pi }{\sqrt{\gamma \beta_{t}}}\right]+1\\ & = & 2(\ln\left[\pi \right]+1). \end{array}$$

This result is well known, and it reflects the relation of the differential Shannon entropies to the coordinate–momentum uncertainty relation. Next, we calculate the electronic entropy from the electronic density. The latter is obtained from a Gaussian integral as

(A13) $$\begin{array}{ccc} \rho^{el}\left(p,t\right) & = & \frac{1}{\pi \sqrt{\beta_{t}\gamma }}\int dP\;e^{-\frac{1}{\beta_{t}}{\left(p+P\right)}^{2}-\frac{p^{2}}{\gamma }}=\frac{1}{\sqrt{\pi \gamma }}\;e^{-\frac{p^{2}}{\gamma }} \end{array}$$

As was discussed above for the total entropy, the quadratic expression for the exponent appearing in the electronic momentum density allows to evaluate the entropy introducing the scaled coordinate $p^{\prime }=\sqrt{\lambda }\;p$ so that the volume element transforms as $dp^{\prime }=dp/\sqrt{\lambda }$. Using Equation (A10), this then leads to

(A14) $$\begin{array}{c} S_{p}^{el}\left(t\right)=\ln\left[\sqrt{\pi \gamma }\right]+\frac{1}{2}. \end{array}$$

In the nuclear case, the density is

(A15) $$\begin{array}{ccc} \rho^{nuc}\left(P,t\right) & = & \frac{1}{\pi \sqrt{\beta_{t}\gamma }}\int dp\;e^{-\frac{1}{\beta_{t}}{\left(p+P\right)}^{2}-\frac{p^{2}}{\gamma }}\\ & = & \frac{1}{\pi \sqrt{\beta_{t}\gamma }}e^{-\frac{P^{2}}{\beta_{t}}}\int dp\;e^{-\frac{\gamma +\beta_{t}}{\beta \gamma }p^{2}-\frac{2P}{\beta_{t}}p}\\ & = & \frac{1}{\pi \sqrt{\beta_{t}\gamma }}\sqrt{\frac{\pi \gamma \beta_{t}}{\gamma +\beta_{t}}}e^{-\frac{P^{2}}{\beta_{t}}}e^{\frac{\beta_{t}\gamma }{\gamma +\beta_{t}}\frac{P^{2}}{\beta_{t}^{2}}}\\ & = & \frac{1}{\sqrt{\pi (\beta_{t}+\gamma )}}e^{-\frac{P^{2}}{\gamma +\beta_{t}}}. \end{array}$$

With the help of Equation (A10), the nuclear entropy is of the form

(A16) $$\begin{array}{c} S_{P}^{nuc}\left(t\right)=\ln\left[\sqrt{\pi (\beta_{t}+\gamma )}\right]+\frac{1}{2}. \end{array}$$

Having calculated the total and single-particle entropies, the mutual information can be determined as

(A17) $$\begin{array}{c} I_{\pi }\left(t\right)=\ln\left[\sqrt{\pi \gamma }\right]+\ln\left[\sqrt{\pi (\beta_{t}+\gamma )}\right]-\ln\left[\sqrt{\gamma \beta_{t}}\pi \right], \end{array}$$

which can be re-written as

(A18) $$\begin{array}{c} I_{\pi }\left(t\right)=\frac{1}{2}\ln\left[1+\frac{\gamma }{\beta_{t}}\right]. \end{array}$$

To determine the covariance in momentum space, we need the expectation values of p,P and the product pP. Regarding the momentum-space density given in Equation (A4), it follows due to symmetry that

(A19) $$\begin{array}{c} {\langle P\rangle }_{t}={\langle p\rangle }_{t}=0. \end{array}$$

This can be traced back to the ansatz of the wave function (Equation (27)), which does not incorporate a mean momentum different from zero. The expectation value of the momentum product is

(A20) $$\begin{array}{ccc} {\langle pP\rangle }_{t} & = & \frac{1}{\pi \sqrt{\beta_{t}\gamma }}\int dP\int dp\;P\;p\;e^{-\frac{1}{\beta_{t}}{\left(P+p\right)}^{2}-\frac{p^{2}}{\gamma }}. \end{array}$$

Introducing the new variable $P^{\prime }=P+p$, the latter integral transforms as

(A21) $$\begin{array}{ccc} {\langle pP\rangle }_{t} & = & \frac{1}{\pi \sqrt{\beta_{t}\gamma }}\int dP^{\prime }\;\int dp\;\left(P^{\prime }p-p^{2}\right)\;e^{-\frac{1}{\beta_{t}}P^{\prime 2}-\frac{p^{2}}{\gamma }}\\ & = & -\frac{1}{\sqrt{\pi \gamma }}\int \;dp\;p^{2}\;e^{-\frac{p^{2}}{\gamma }}=-\frac{1}{2}\gamma , \end{array}$$

where we employed symmetry in the integration over $P^{\prime }$, and we used the analytical result for an Gaussian integral as $\int dy\;y^{2}\;e^{-y^{2}}=\sqrt{\pi }/2$. We then arrive at the result

(A22) $$\begin{array}{c} cov_{\pi }\left(t\right)=-\frac{1}{2}\gamma . \end{array}$$

Finally, to calculate the correlation, we need the variances in the two momentum variables. From Equation (A21) we realize that

(A23) $$\begin{array}{c} {\langle p^{2}\rangle }_{t}=\frac{\gamma }{2}. \end{array}$$

In calculating the variance for the variable *P*, again the transformation $P^{\prime }=P+p$ is used and properties of integrals over Gaussians are employed. One finds

(A24) $$\begin{array}{ccc} \langle P^{2}\rangle & = & \frac{1}{\pi \sqrt{\beta_{t}\gamma }}\int dP\;\int dp\;P^{2}\;e^{-\frac{1}{\beta_{t}}{\left(P+p\right)}^{2}-\frac{p^{2}}{\gamma }}\\ & = & \frac{1}{\pi \sqrt{\beta_{t}\gamma }}\int dP^{\prime }\;\int dp\;{\left(P^{\prime }-p\right)}^{2}\;e^{-\frac{1}{\beta_{t}}P^{\prime 2}-\frac{p^{2}}{\gamma }}\\ & = & \frac{1}{\pi \sqrt{\beta_{t}\gamma }}\int dP\;\int dp\;\left(P^{2}+p^{2}\right)\;e^{-\frac{1}{\beta_{t}}P^{2}-\frac{p^{2}}{\gamma }}\\ & = & \frac{1}{\sqrt{\pi \beta_{t}}}\int dP\;P^{2}\;e^{-\frac{1}{\beta_{t}}P^{2}}+\frac{1}{\sqrt{\pi \gamma }}\int \;dp\;p^{2}\;e^{-\frac{p^{2}}{\gamma }}\\ & = & \frac{1}{2}\left(\beta_{t}+\gamma \right), \end{array}$$

so that

(A25) $$\begin{array}{c} \sigma_{P}^{2}=\frac{1}{2}\left(\beta_{t}+\gamma \right). \end{array}$$

Having calculated the variances, one arrives at an expression for the correlation, which reads

(A26) $$\begin{array}{c} corr_{\pi }\left(t\right)=-\frac{1}{\sqrt{1+\beta_{t}/\gamma }}. \end{array}$$

## Appendix B. Entropies for Strong Coupling

Here, the entropies for the strong coupling case are determined analytically using the ansatz of Equation (47) for the wave function. Using the nuclear density

(A27) $$\begin{array}{c} \rho^{nuc}\left(R,t\right)=\sqrt{\frac{\pi }{\beta_{t}}}\;e^{-\beta_{t}{\left(R-R_{t}\right)}^{2}}, \end{array}$$

the associated entropy is, using the result of Equation (A10),

(A28) $$\begin{array}{c} S_{x}^{nuc}\left(t\right)=\ln\left[\sqrt{\frac{\pi }{\beta_{t}}}\right]+\frac{1}{2}. \end{array}$$

With the electronic density

(A29) $$\begin{array}{c} \rho^{el}\left(r,t\right)=\sqrt{\frac{\gamma }{\pi }}\;e^{-\gamma {\left(r-R_{0}\right)}^{2}} \end{array}$$

an equivalent calculation as performed in the nuclear case yields the electronic entropy

(A30) $$\begin{array}{c} S_{x}^{el}\left(t\right)=\ln\left[\sqrt{\frac{\pi }{\gamma }}\right]+\frac{1}{2}. \end{array}$$

The same result is obtained in the BO case for β→∞, which means that the nuclear wave function is strongly localized so that the *R*-dependence of the electronic wave function can be neglected and one recovers the diabatic case.

For the present situation of a separable wave function, the total entropy is just the sum of the single-particle entropies, and it reads

(A31) $$\begin{array}{c} S_{x}\left(t\right)=\ln\left[\frac{\pi }{\sqrt{\beta_{t}\gamma }}\right]+1. \end{array}$$

Because of the Fourier properties of Gaussians, the nuclear momentum density has the same functional form as the coordinate density upon the replacements R→P and $\beta_{t}\to 1/\beta_{t}$. This leads to the entropy

(A32) $$\begin{array}{c} S_{P}^{nuc}\left(t\right)=\ln\left[\sqrt{\pi \beta_{t}}\right]+\frac{1}{2}. \end{array}$$

Using the replacement r→p,γ→1/γ yields

(A33) $$\begin{array}{c} S_{p}^{el}\left(t\right)=\ln\left[\sqrt{\pi \gamma }\right]+\frac{1}{2}, \end{array}$$

and the total entropy is additive:

(A34) $$\begin{array}{c} S_{\pi }\left(t\right)=\ln\left[\sqrt{\gamma \beta_{t}}\pi \right]+1. \end{array}$$
